# Preeclampsia and maternal breast cancer risk by offspring gender: do elevated androgen concentrations play a role?

**DOI:** 10.1038/sj.bjc.6603921

**Published:** 2007-08-07

**Authors:** R Troisi, K E Innes, J M Roberts, R N Hoover

**Affiliations:** 1 Division of Cancer Epidemiology and Genetics, National Cancer Institute, Bethesda, MD, USA; 2 Department of Community and Family Medicine, Dartmouth Medical School, Hanover, NH, USA; 3 Center for the Study of Complementary and Alternative Therapies, University of Virginia Health System, Charlottesville, VA, USA; 4 Magee Womens Hospital, University of Pittsburgh, Pittsburgh, PA, USA

**Keywords:** preeclampsia, androgens, hormones, breast cancer, maternal, offspring gender

## Abstract

Among older mothers, preeclampsia in the first pregnancy was associated with a reduction in maternal breast cancer risk that was significantly more pronounced in women bearing male than female infants. Androgen concentrations in male, preeclamptic pregnancies were consistent with the hypothesis that elevated pregnancy androgens might mediate this apparent modifying effect of fetal gender.

The reduced risk of breast cancer observed in mothers subsequent to preeclamptic pregnancies (Polednak and Janerich, 1983; Thompson *et al*, 1989; Troisi *et al*, 1998; Vatten *et al*, 2002; Innes and Byers, 2004; Aagaard-Tillery *et al*, 2006) is curious and the mechanisms mediating it remain largely unexplored. In their recent paper on preeclampsia/pregnancy-induced hypertension and maternal breast cancer risk, Vatten *et al* (2007) further characterise this association by offspring's gender, reporting a risk reduction with preeclampsia only when the mother carries a male fetus. The authors conclude that their findings are consistent with a protective effect originating with exposures incurred during the pregnancy rather than to an underlying biological trait of the mother, presumably because such factors would be unrelated to whether she conceives a female or male child. Previous studies (Martin *et al*, 1986; Steier *et al*, 2002; Troisi *et al*, 2003; Atamer *et al*, 2004) indicate elevations in third trimester circulating maternal androgen concentrations in preeclampsia compared with uncomplicated pregnancies. Maternal exposure to elevated androgen concentrations has been hypothesised to mediate the protective influence of preeclampsia, perhaps by antagonising oestrogen's effects on breast epithelial cells (Innes and Byers, 1999; Cohn *et al*, 2001).

We attempted to replicate the findings of Vatten *et al* of greater maternal breast cancer protection associated with male preeclamptic pregnancies using linked birth and tumour registry data from a previous population-based study of early-onset breast cancer (Innes and Byers, 2004). To address the possible role of androgen concentrations in mediating this apparent risk reduction, we re-examined data from a longitudinal study of preeclampsia (Troisi *et al*, 2003).

## MATERIALS AND METHODS

### New York state-linked birth and tumour registry data

The study population in this matched case–control study comprised primiparous women with singleton births who completed a first pregnancy in New York state (NY) after 1977 (for detailed description of study population and methods, see Innes and Byers, 2004). To ensure comparability to Vatten *et al*'s study, we included only women with singleton first pregnancies in the present analysis. Cases were 2489 primiparous women who were diagnosed with breast cancer in NY between 1979 and 1995 and at least 1 year after completion of the first pregnancy; age at diagnosis ranged from 22 to 55 years. Controls were 9967 primiparous women matched to cases on county of residence and delivery date and who were not diagnosed with breast or endometrial cancer in NY. Information on factors characterising each woman's first pregnancy was obtained from the birth record of her first-born infant. The association of preeclampsia and maternal breast cancer risk was evaluated using conditional logistic regression (Breslow and Day, 1980).

### University of Pittsburgh preeclampsia study

Data were from a longitudinal preeclampsia study conducted at the University of Pittsburgh (Pittsburgh, PA, USA) that included all women who attended the Magee Womens Hospital's obstetric practice, delivered between February 1994 and May 1998 and agreed to participate. Eligible for analysis were 86 cases and 86 controls from a previous case-control study of preeclampsia with singleton pregnancies and without pre-existing diabetes or hypertension (Troisi *et al*, 2003). Informed consent for a questionnaire, interview and blood collection was obtained from all study participants. Information on the pregnancy was obtained from medical records. Preeclampsia was defined by explicit criteria (described in Troisi *et al*, 2003) based on blood pressure measurements, proteinuria and hyperuricemia (Chesley, 1985), and all diagnoses were reviewed by a jury of clinical experts.

Maternal blood was collected at admission for labor and delivery. The hormone assays have been described (Troisi *et al*, 2003). Briefly, unconjugated concentrations of androstenedione and testosterone were measured in serum by radioimmunoassay. The coefficients of variation based on blinded quality control samples were 10.2% for androstenedione and 9.6% for testosterone.

The hormones were logarithmically transformed to remove skewness from their distributions. Mean maternal androgen concentrations were determined for preeclampsia and uncomplicated pregnancies by offspring gender using analysis of covariance, and geometric means and 95% confidence intervals are presented. Per cent difference was derived from the beta estimate of the case/control (dichotomous) variable in a generalised linear regression model with androgens as the dependent variables. Interactions were tested in linear regression models, with androgens as the dependent variables and case/control status, offspring gender and a product term for case/control status and offspring gender as the independent variables. Statistical significance was defined as *P*<0.05 (two-sided).

## RESULTS

### New York state-linked birth and tumour registry data

Preeclampsia was associated with a modest, non-significant maternal breast cancer risk reduction overall (maternal age-adjusted OR=0.87) that was similar to Vatten *et al*'s (2007) overall estimate (RR=0.86), but a marked and significant risk reduction among women who delivered their first infant after the age of 30 years (adjusted OR=0.33) (Table 1). The overall association of preeclampsia to subsequent breast cancer risk did not differ by offspring gender (adjusted OR=0.86 *vs* 0.90 for women who delivered a son or a daughter, respectively). However, among the mothers older than age 30, the risk reduction associated with preeclampsia was significantly greater for those who carried a male infant (adjusted OR=0.18) than female infant (adjusted OR=0.52) (*P* for interaction=0.01) (Table 1), although risk reductions were observed for pregnancies involving either gender.

### University of Pittsburgh preeclampsia study

While mean androstenedione and testosterone concentrations were significantly higher in both male and female preeclamptic compared with uncomplicated pregnancies, absolute concentrations in both preeclamptic and uncomplicated pregnancies were significantly higher in those involving males than females (Table 2). Indeed, the concentrations in the uncomplicated male pregnancies (382 for androstenedione and 166 for testosterone) were similar to those in preeclamptic female pregnancies (381 and 161, respectively).

## DISCUSSION

The overall estimates for preeclampsia and breast cancer risk in the present study were similar whether the pregnancy involved a male or female fetus, consistent with findings from a recent case–control study conducted in Long Island, NY (Terry *et al*, 2007). However, among women who were older at first birth (>30 years), the risk reduction associated with a male fetus was significantly greater than that associated with a female fetus.

Previous mechanistic investigations regarding the association of preeclampsia with breast cancer risk have focused on hormones with an established role in breast carcinogenesis, including oestrogens (Tamimi *et al*, 2003; Troisi *et al*, 2003) and androgens (Troisi *et al*, 2003). If the findings of Vatten *et al* (2007) and what we observe in the present study among older mothers of male preeclamptic pregnancies are real, characterising the hormonal profile of preeclamptic pregnancies by fetal gender and maternal age may provide additional insight into the mechanisms underlying the apparent protective effect of preeclampsia.

In the present study, the highest androgen concentrations were observed in male, preeclamptic pregnancies. Consistent with these findings, Steier *et al* (2002) also showed elevated testosterone concentrations in male preeclamptic pregnancies relative to both female preeclamptic pregnancies and uncomplicated pregnancies; in contrast to our observations, the per cent difference between testosterone in the preeclamptic and uncomplicated pregnancies was higher in male- than in female-bearing pregnancies, perhaps reflecting differences in study sampling times. Collectively, these data indicate higher absolute (and in Steier *et al*'s data, higher relative) androgen concentrations in the maternal circulation of preeclamptic pregnancies involving a male fetus compared with those involving a female fetus. This suggests that the observed protective effect of preeclampsia on breast cancer risk may in part rely on meeting or exceeding a specific threshold of exposure to endogenous androgens. Because uncomplicated male pregnancies have higher androgen concentrations than uncomplicated female pregnancies, one might expect offspring gender to be associated with maternal breast cancer risk even in normal pregnancy. However, previous studies have not consistently demonstrated such an association (Olsen and Storm, 1998; Troisi *et al*, 1998; Hsieh *et al*, 1999; Cnattingius *et al*, 2005); thus, if protection is associated with absolute androgen concentration, the threshold for this effect must be above the levels characterising uncomplicated male pregnancies. Whether this is biologically plausible is unclear.

Preeclamptic pregnancies that involve male offspring may also differ in other biological factors, such as human chorionic gonadotropin (hCG) (Steier *et al*, 2002), which has been shown to be higher in male preeclamptic pregnancies than in female preeclamptic pregnancies or in uncomplicated pregnancies involving either offspring gender. Human chorionic gonadotropin has been linked to reduced breast cancer risk in human populations, and has been repeatedly shown to promote mammary gland differentiation and inhibit neoplastic changes in animal models (Innes and Byers, 1999; Janssens *et al*, 2007). The significantly greater increases in hCG associated with male preeclamptic pregnancies may also in part account for the higher androgen concentrations observed in both our data and that of Steier *et al* (2002). Other biological mechanisms have also been proposed to explain the observed protective effect of preeclampsia, including immunological factors (Polednak and Janerich, 1983) and alterations in IGF-1 and other compounds linked to breast cancer initiation and promotion (Innes and Byers, 1999, 2004). More recent speculation has focused on the antiangiogenic profile of women who develop pregnancy-induced hypertension (Aagaard-Tillery *et al*, 2006).

The possibility that preeclampsia affords greater protection for maternal breast cancer in pregnancies involving male fetuses merits replication in other large population-based studies. If confirmed, evaluation by offspring gender of the biomarkers mentioned above as well as others that are known to be altered in preeclamptic pregnancies may further our understanding of the apparent protective influence of preeclampsia on breast carcinogenesis.

## Figures and Tables

**Table 1:** Odds ratios (OR) and 95% confidence intervals (CI) for preeclampsia (PE) in the first pregnancy and subsequent maternal breast cancer risk among women 22–55 years of age, New York state, USA

	**No. of cases/controls with PE**	**OR^a^**	**95% CI**
*All births*	95/386	0.87	(0.67–1.13)
Male infant	45/208	0.86	(0.60–1.22)
Female infant	50/178	0.90	(0.64–1.27)
*p* interaction 0.86			
			
*Maternal age* >*30*	46/79	0.33	(0.16–0.65)
Male infant	22/42	0.18	(0.04–0.87)
Female infant	24/37	0.52	(0.13–2.0)
*p* interaction 0.01			

a Odds ratio adjusted for maternal age.

**Table 2:** Means for and per cent difference (% diff) with 95% confidence interval (CI) in maternal androgen concentrations between preeclampsia (PE) cases and uncomplicated (control) pregnancies adjusted for weeks of gestation, by offspring gender

	**Offspring gender**								
	**Female**				**Male**				
**Hormone**	**PE**	**Control**	**% diff^a^**	**95% CI**	**PE**	**Control**	**% diff^a^**	**95% CI**	***p* interaction^b^**
Androstenedione (ng dl^−1^)	381	320	27.3	15.6, 40.1	425	382	12.8	4.7, 21.6	0.04
Testosterone (ng dl^−1^)	161	139	28.5	16.0, 42.4	180	166	11.4	2.5, 21.2	0.03

a Per cent difference (% diff) derives from the beta estimate of the case/control variable in a generalised linear regression model, with logarithm-transformed values for the maternal androgens as the dependent variables. Interactions were tested in a linear regression model, with logarithm-transformed androgens as the dependent variables and case/control status, offspring sex and an interaction term for case/control status × offspring sex as the independent variables.

b *p* interaction refers to the comparison of per cent difference in each androgen between PE and control pregnancies by offspring gender.
